# Cu- and Ag-mediated inactivation of *L. pneumophila* in bench- and pilot-scale drinking water systems

**DOI:** 10.1128/aem.01073-24

**Published:** 2024-12-18

**Authors:** Chelsea L. Hintz, Brian Morris, Sue Witt, Nicole Sojda, Helen Y. Buse

**Affiliations:** 1 Environmental Protection Agency, Office of Research and Development, Center for Environmental Solutions and Emergency Response 96850 , Cincinnati, Ohio, USA; 2 Pegasus Technical Services, Inc., c/o U.S. EPA, Cincinnati, Ohio, USA; 3 Aptim Environmental Services, c/o U.S. EPA, Cincinnati, Ohio, USA; Norwegian University of Life Sciences, Ås, Norway

**Keywords:** environmental monitoring, copper (Cu) and silver (Ag) ions, disinfection, potable water, premise plumbing system

## Abstract

*Legionella pneumophila* (Lp) is an opportunistic drinking water pathogen that can cause infections through the inhalation of Lp-containing aerosols and can occur in premise plumbing systems. In this work, the use of copper (Cu) and silver (Ag) ions was evaluated at the bench and pilot scale to determine (i) the effective independent concentrations of copper and silver that are efficacious in inactivating Lp, (ii) the impact of various water quality parameters on the efficaciousness of copper and silver ions, and (iii) the effectiveness and practicality of using dissociation to produce ions at the pilot scale. At the bench scale, it was determined that 0.3 ppm and 0.03 ppm of Cu and Ag, respectively, achieved 6-log inactivation of Lp in 5 h in experimental buffer. But, in dechlorinated filter-sterilized tap water, the same concentrations of Cu were not effective, and the effectiveness of Ag was slower. pH and dissolved inorganic carbon content were found to be important parameters in determining if the use of Cu and Ag ions is appropriate. At the pilot scale, dissociation was successfully used to produce Cu and Ag ions. Target levels of ions were met at the pilot scale but were difficult to achieve, and no impact was observed on Lp concentrations. Results from this study suggest that there are important caveats in the application of this technology when applied in a drinking water matrix and prior understanding of a system’s water chemistry may be important to determine the effectiveness of Lp disinfection using Cu and Ag.

**IMPORTANCE:**

This work sheds light on the effectiveness of using Cu and Ag ions to inactivate (or kill) *Legionella pneumophila. Legionella* is an opportunistic drinking water pathogen of public health concern. This work demonstrates that there are important caveats in the application of using Cu and Ag ions to inactivate *Legionella pneumophila*.

## INTRODUCTION

*Legionella pneumophila* (Lp) is an opportunistic waterborne pathogen and is ubiquitous in the environment. Lp can be found in drinking water systems where they eventually colonize and become a public health risk within premise plumbing systems (or building water systems) (1, 2). Lp is a significant public health concern, particularly for immunocompromised individuals, due to increasing incidence rate of legionellosis in the United States (3) and Europe (4). Infections are primarily caused by the inhalation of Lp-containing aerosols generated from fixtures and devices such as showerheads and sink faucets and cooling towers (2).

Lp can survive under a wide range of environmental conditions which can make it difficult to eliminate Lp from premise plumbing systems. Lp has been detected at a wide range of temperatures (19°C–44°C) (5) with levels increasing under warmer temperatures (35℃–37°C) (5, 6). pH can also vary across premise plumbing systems with Lp survival being observed at pH 5.0 to 8.5 (7). Lp is a facultative anaerobe and can survive in biofilms (sessile form) and in bulk water (planktonic form) and can parasitize amoeba hosts for amplification (2) further complicating control of Lp. Lp can also survive for long periods of time in nutrient-poor environments (such as sterile distilled water, in both sessile and planktonic forms), but reproduction may be limited (1, 8, 9). Lastly, there are other clinically relevant drinking water pathogens such as *Pseudomonas aeruginosa* which can survive under similar environmental conditions as Lp (10).

Methods to control Lp within premise plumbing systems can include thermal inactivation, filtration, ozonation, ultraviolet (UV) light, chlorine-based methods, or a combination of methodologies. Another treatment technique is the addition of copper (Cu) and silver (Ag) ions to premise plumbing systems, particularly hot water recirculation systems, to inactivate (or kill) Lp. The use of Cu and Ag ions has also been used to control *P. aeruginosa* (11). The use of Cu and Ag has been tested in both laboratory settings (12–15) and hospital systems (16–26). In lab and bench-scale studies, the production of Cu and Ag ions is often through a dissociation methodology (e.g., dissolving copper sulfate [CuSO_4_] and silver nitrate [AgNO_3_] in water), while at building-scale (such as hospitals), Cu and Ag ions are produced electrolytically via commercially available units. Pilot-scale studies (11, 15, 27) have also utilized commercial units. The proposed mechanisms of Cu and Ag bactericidal properties are cellular lysis caused by cupric (Cu^2+^) and silver (Ag^+^) ion-mediated disruption of the cell wall and disruption of cellular respiration and DNA translation (12, 22). This mechanism relies on Cu^2+^ and Ag^+^ staying in solution and not forming complexes (14, 28). However, water quality parameters such as pH, total organic carbon (TOC), and phosphate have been shown to influence the effectiveness of Cu and Ag ions (14, 26, 28). Ionic copper, particularly, can be difficult to keep in solution and is influenced by pH, TOC, and orthophosphate (28, 29). Lastly, the presence of Cu^2+^ can potentially enhance the formation of disinfection byproducts (DPBs) (particularly trihalomethanes) during chlorination (30) and could be problematic if concentrations of DPBs exceed regulatory levels.

Prior work has shown the effectiveness of Cu and Ag under controlled lab conditions (12) which can avoid the influence of various water chemistry parameters as described above. However, the effectiveness of Cu and Ag ions as bactericidal agents has been mixed under “real-world” conditions at the pilot and building scale (21) and ranges from no success (16) to complete eradication (20) for both Lp (16, 20, 21) and *P. aeruginosa* (11). Furthermore, ion concentrations can be controlled under ideal lab conditions, while maintenance of ion concentrations within larger systems using commercial copper-silver ionization (CSI) units is difficult and may result in exceedances above recommended levels of Cu and Ag by regulatory agencies (26, 31). The complexities of premise plumbing systems such as varying temperatures, water chemistry, plumbing materials, and ion concentrations (depending on where the CSI unit is installed) (24, 26, 31) all add to the difficulty of generating and maintaining ion levels within these systems.

Thus, there is a need for research that combines both lab and pilot-scale experiments and consistent methodologies for Cu and Ag ion generation to better understand their microbicidal efficacy. In this study, benchtop experiments evaluated the inactivation of planktonic Lp using either Cu and Ag ions generated by a dissociation method and allowed for direct control of various water quality parameters. Lastly, we evaluated the effectiveness and practicality of using a commercially available CSI unit versus a dissociation methodology to produce Cu and Ag ions in a complex pilot-scale pipe loop using utility-supplied water and naturally occurring Lp to reflect a real-world application of this methodology. To our knowledge, there has yet to be a study that evaluates whether a dissociation methodology can be utilized within a pilot-scale pipe loop at this scale.

## MATERIALS AND METHODS

### Bench-scale experiments

#### Bacterial culture preparation

A Lp serogroup-1 drinking water isolate (previously stored at −80°C in buffered yeast extract [BYE] broth [Fisher Scientific] with 20% [vol/vol] glycerol [Fisher Scientific]) was used to conduct the bench-scale experiments (sg1-Oh in reference 32). The stock culture was defrosted, and 5 µL was spread-plated onto commercially available buffered charcoal yeast extract (BCYE) agar (Thermo Scientific) and incubated in ambient air at 37°C for 3 days. A Lp colony was inoculated in 10 mL of BYE broth and grown overnight with continuous shaking at 37°C. Growth was observed in the overnight culture, and 1.2 mL was transferred to microcentrifuge tubes and centrifuged at room temperature for 4 min at 7,000 rpm and washed four times with experimental buffer (either DIC10 or DF Tap Water, described below). The final concentration of Lp was determined to be 10^7^ colony-forming unit (CFU) mL^−1^ as described below.

#### Experimental treatments and preparation

**Fig 1:**
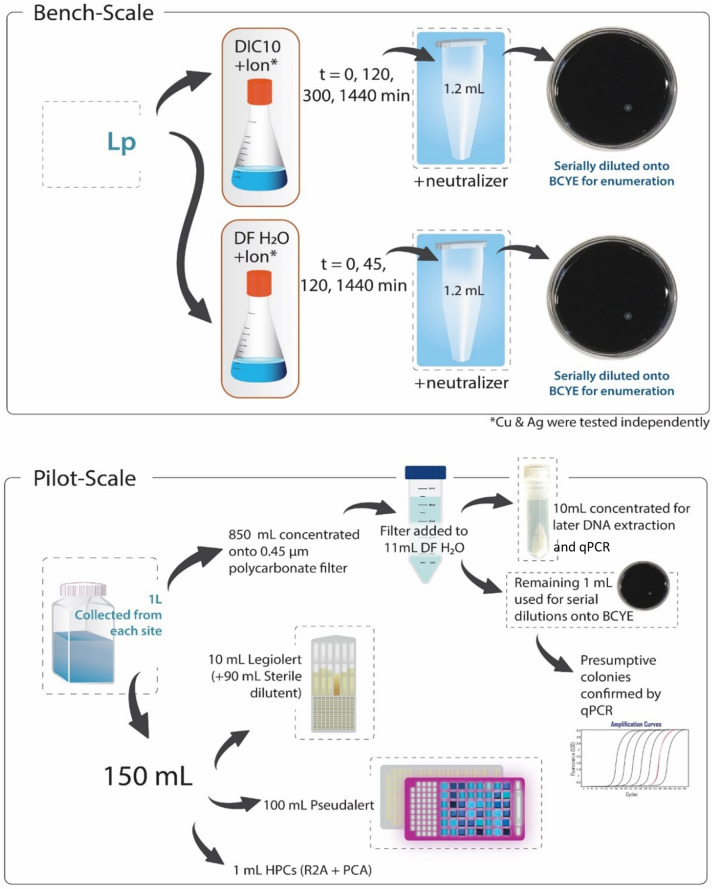
Methods summary diagram for the bench- and pilot-scale experiments.

The bench-scale experiments were conducted in two test media: a solution that contained 10 mg L^−1^ of dissolved inorganic carbon content (hereafter referred to as DIC10) and dechlorinated, filter-sterilized tap water (hereafter referred to as DF Tap Water) (Fig. 1). DIC10 was made by dissolving sodium bicarbonate into deionized water and adjusting the pH to 8 which ensured that both Cu and Ag ions would stay in solution and not form complexes (29). DF Tap Water was chosen as a test media to represent drinking water typically found in premise plumbing systems (in Ohio, USA). Tap water was collected and dechlorinated under UV light until the total chlorine residual (determined via Hach kits, described below) was below detectable levels and then filtered through a 0.45-µm polycarbonate filter (Pall Life Sciences, Nassau, NY, USA) for sterility. DF Tap Water had a pH of 8.2–8.3 and on average had a total organic carbon content of <1.5 mg L^−1^. Water was dechlorinated to isolate the impact of Cu and Ag ions on inactivation.

We conducted individual experiments for Cu and Ag using dissociation to produce ions. Target concentration for Cu was 0.3 ppm, and target concentration for Ag was 0.03 ppm based on previous literature values (13, 14, 21, 22, 33–35). Experiments were conducted in both DIC10 and DF Tap Water. Cu solution was prepared by making a 2% CuSO_4_ (Sigma Aldrich) solution in Milli-Q water, and Ag solution was prepared by making a 0.5% AgNO_3_ (Sigma Aldrich) solution in Milli-Q. Both solutions were then diluted to the target concentration using experimental buffer (either DIC10 or DF Tap Water) in high-density polyethylene (HDPE) bottles. Ion solutions were freshly prepared on the day of the respective experiment. Samples were analyzed by inductively coupled plasma spectrometry (ICP-MS; 33) to quantify ion concentrations (both total and dissolved) at the beginning and end of each experiment. Ten percent of sodium thiosulfate (Fisher Scientific) was used as a neutralizing agent to stop the bactericidal activity of Cu and Ag ions in our treatments at individual timepoints and was used in our control treatments (described below).

Experiments were conducted at room temperature in 125-mL polycarbonate flasks, and 0.5 mL of 10^7^ CFU mL^−1^ Lp culture was added to 49.5 mL of either Cu or Ag solution (“Cu treatment” or “Ag treatment”) giving a final Lp concentration of 10^5^ CFU mL^−1^. Four replicates were used. For the DIC10 experiments, samples were collected at 0, 120, 300, and 1440 min, and for DF Tap Water experiments, samples were collected at 0, 45, 120, and 1440 min (except for the Ag treatment at 120 min). For the DF Tap Water experiments, an additional 45-min timepoint was added (and the 300-min timepoint removed) so as to not miss the inactivation curve. At each timepoint, 1.2 mL was removed from each flask and placed into a 1.7 microcentrifuge tube containing 12 µL of neutralizer. Samples were then serially diluted with experimental buffer, 100 µL plated onto BCYE agar (Thermo Scientific), and enumerated after incubation at 36°C for 4 days.

Appropriate controls were utilized and consisted of a sterile control for each ion solution (“Cu control” or “Ag control”), Lp in experimental buffer only (“control”) (three replicates), a neutralization control for each ion where 0.5 mL of neutralizer was added to 49.5 mL of ion solution and then 0.5 mL of Lp culture was added (“Cu neutralized” or “Ag neutralized”) (three replicates per ion), and a neutralized Lp control (“neutralized”) that consisted of 0.5 mL neutralizer solution added to 49.5 mL experimental buffer and then 0.5 mL Lp culture was added.

### Pilot-scale experiments

**Fig 2:**
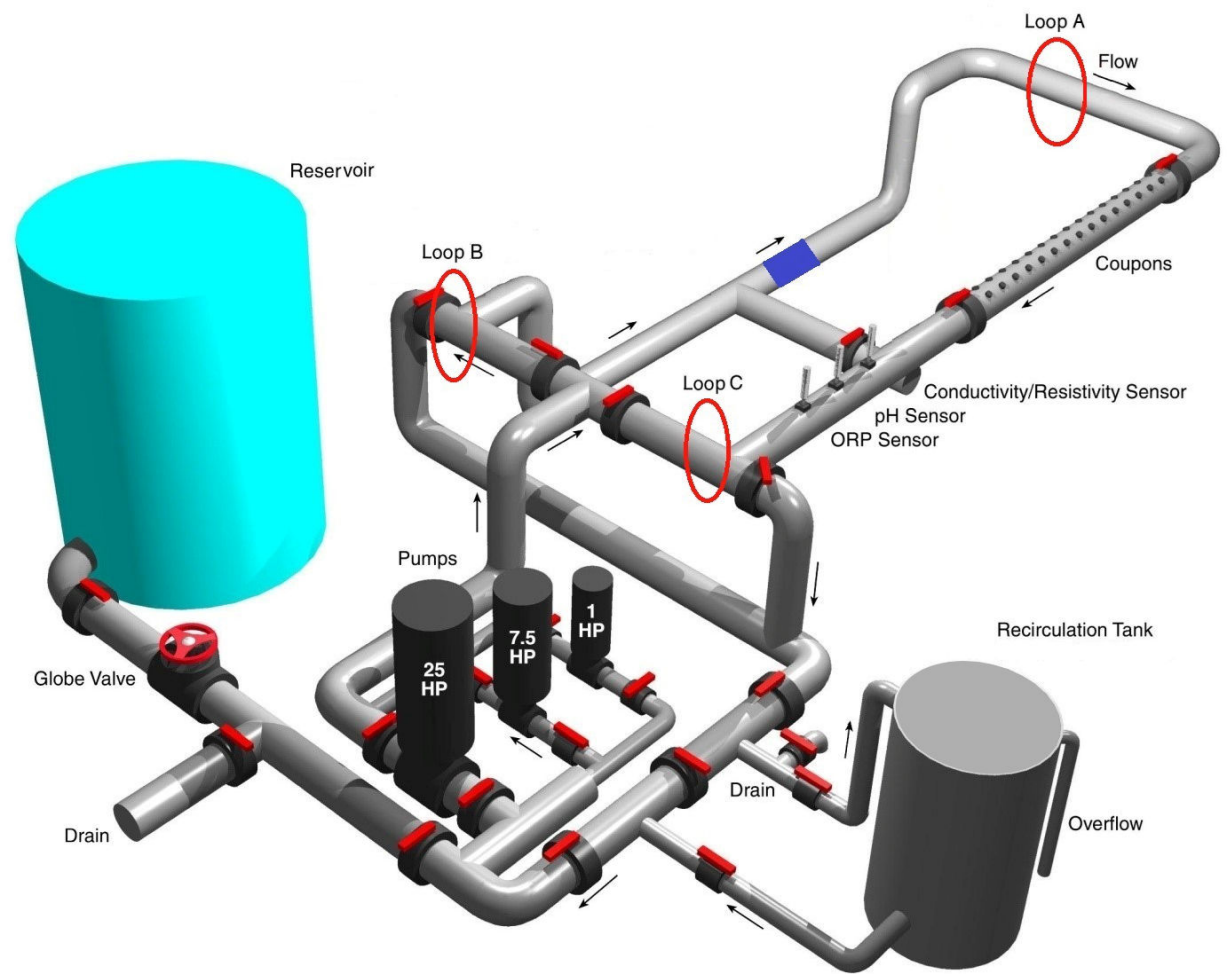
Map of the Drinking Water Distribution System Simulator. Arrows represent the direction of water flow. The blue box represents the commercial CSI unit that was temporarily installed. Five sites were sampled throughout the DSS. Cu and Ag ions were added into the recirculation tank. Adapted from Szabo et al. (37) with permission of the publisher.

Two pilot-scale experiments were conducted at the EPA Testing & Evaluation Facility in Cincinnati, OH, using a Drinking Water Distribution System Simulator (DSS). The DSS (Fig. 2) is constructed of polyvinyl chloride (PVC) pipe and recirculates approximately 833 L (or 220 gallons). The main components of the DSS are a large reservoir to supply water to the PVC pipe loop, which is approximately 23 m (or 75 feet) of 6-inch diameter PVC pipe (except for a section that is 4-inch diameter and 1.5 m [or 5 feet] long), a recirculation tank (378 L or approximately 100-gallon capacity), water pumps, and the associated valves and electronic control devices necessary to operate the system. Operation of the DSS system with the recirculation tank in-line enables introduction of any microbial and/or chemical additive to become thoroughly mixed in a few minutes within the main pipe loop. New water is added to the DSS (and also discharged) at a rate of approximately 48 L h^−1^ to maintain the chlorine residual. The DSS can also be run in recirculation mode where no water is added or lost from the system. Lp naturally occurs within the DSS, and therefore the DSS was not inoculated (36).

There are five sampling points within the DSS (Fig. 2): the reservoir (Res) which serves as the source of new water and is not recirculated, the recirculation tank (Tank), and at three points throughout the DSS (Loops A, B, and C). Additionally, a calibrated multiparameter sonde (Horiba, Kyoto, Japan) was placed in the recirculation tank to monitor pH, conductivity, temperature, and oxidation-reduction potential (ORP). For each experiment, background samples for all analytes were collected prior to the addition of Cu and Ag ions.

#### Production of Cu and Ag ions

For the first pilot-scale experiment, a commercially available CSI unit was installed in-line on the DSS (represented by the blue square in Fig. 2), and Cu and Ag ions were produced via electrolysis. Briefly, the CSI unit alternated between passing electric current through two separate metal bars containing a heterogeneous mixture of metals, including Cu and Ag. The production of Cu and Ag ions and associated CSI unit settings were set and monitored by the commercial vendor. Target concentrations for the electrolysis experiment were 0.4 ppm for Cu and 0.04 ppm for Ag as recommended by the manufacturer. The CSI unit was removed from the DSS after the completion of this experimental phase (6 weeks).

For the second pilot-scale experiment, Cu and Ag ions were produced via dissociation using CuSO_4_ and AgNO_3_, respectively over a period of 10 weeks. Target concentrations for the dissociation experiment were 0.4 ppm for Cu and 0.04 ppm for Ag to match the experiment using the commercial CSI unit. For each ion, concentrated stock solutions were made separately by dissolving the respective reagent into reagent grade water (Milli-Q, Millipore Sigma, MA, USA). Concentrations of the stock solutions were 796 ppm for Cu and 31.75 ppm for Ag. Stock solutions were then pumped directly into the recirculation tank via separate chemical metering pumps (LMI, Ivyland, PA, USA). To initially reach targeted concentrations, we set the DSS to recirculation mode and added 418 mL of Cu stock solution and 1,049 mL of Ag stock solution over a period of 1 h. After 24 h, refresh water was allowed to enter the DSS again, and we began a constant drip of Cu (initial rate of 0.4 mL min^−1^) and Ag (initial rate of 1 mL min^−1^) solutions to maintain our target concentrations. A constant drip of Cu and Ag ions was maintained for the duration of the experiment. Flow rates of Cu and Ag ions were periodically checked and were adjusted periodically to reach or maintain our target concentrations.

Cu and Ag concentrations were monitored via ICP-MS analysis (38) in both experiments and via Hach colorimetric kits (Cu, Hach Method 8506; Ag, Hach Method 8120) for the dissociation experiment. ICP samples were routinely taken from the Tank, Loop A, Loop B, and Loop C. ICP samples were collected intermittently from Res to serve as a negative control since little to no Cu and Ag were expected to be in the supplied utility water. Samples for Hach colorimetric analysis were collected from Tank and Loop A and were used as an immediate quantification of Cu and Ag ion concentrations.

#### Water chemistry

In the Cu Ag dissociation pilot-scale experiment, samples were routinely collected from five locations within the DSS (Fig. 2) for water chemistry measurements and included free and total chlorine (Hach Methods 8021 and 8167, respectively), temperature, and hardness (Hach Model HA-71A). Total organic carbon was collected from Loop A, while conductivity (µS cm^−1^), ORP (mV), and pH were collected at the Res site in the DSS. Samples for TOC were acidified with phosphoric acid and were quantified following EPA Method 415.3 rev 1.1 (39). Samples were collected for the quantification of disinfection byproducts, specifically total trihalomethanes (TTHMs) and haloacetic acids (HAA5s), during week 4 and week 10 and were processed according to EPA Method 524.2 (40) and EPA Method 552.2 (41), respectively.

#### Microbial analyses

In order to quantify the impact of Cu and Ag ionization on the microbial community within the DSS, bulk water samples were routinely collected from five locations within the DSS. One liter of samples was collected using autoclave-sterilized HDPE bottles, and chlorine was neutralized with 1 mL of 10% sodium thiosulfate solution. At locations Loop A, B, and C, the sampling port was flushed for 10 seconds before a sample was collected. Upon return to the lab, 850 mL of each sample was filtered through a 0.45-um polycarbonate filter (Pall Life Sciences, Nassau, NY, USA) (Fig. 1). Filters were then placed into a 50-mL conical tube with 11-mL dechlorinated filter-sterilized tap water (DF Tap Water) and vortexed on high for 30 seconds (~8-fold concentration of the original bulk water sample). For three of the five sampling locations (Res, Tank, and Loop B), 1 mL of the concentrated sample was transferred to a microcentrifuge tube and used for colony-forming unit enumeration. The remaining 10 mL of concentrated sample was processed as described in “DNA extraction,” below.

We targeted *Legionella* within the DSS by spread-plating concentrated bulk water samples onto BCYE agar as described previously (36). A subsample was heat-treated (30 min at 50°C) to reduce non-*Legionella* bacteria (36), and then 10-fold dilutions were prepared of heat-treated and non-heat-treated samples using DF Tap Water and plated on BCYE. BCYE was incubated at 36°C for 5 days (42). After incubation, presumptive *Legionella* colonies were counted, and a representative colony was selected for confirmation by qPCR as described below.

The remaining unconcentrated bulk water sample was used for quantification of Lp (Legiolert, Idexx, Westbrook, ME, USA; the upper limit of detection (LOD) is 2272.6 Most Probable Number (MPN) 100 mL^−1^) and *Pseudomonas aeruginosa* (Pseudalert, Idexx, Westbrook, ME, USA; the upper LOD is 2419.6 MPN 100 mL^−1^) following manufacturer’s protocols. Legiolert and Pseudalert tests detect an organism-specific bacterial enzyme and are culture-based assays. These tests were included since they are marketed as a simple laboratory method compared to the gold standard culture method, and there has been increasing adoption of these methods as they are marketed as easy to use. Therefore, we wanted to evaluate the results between Legiolert and the BCYE culture method. One milliliter of the bulk water sample was also analyzed for heterotrophic plate counts (HPCs) on Reasoner’s 2A (R2A) (BD Difco) and plate count (PC) agar (BD Difco). R2A was incubated at 28°C for 7 days, and PC was incubated at 36°C for 48 h (43). Positive Legiolert wells were randomly sampled from each sample tray for later confirmation via qPCR (described below).

#### DNA extraction

For all five sampling locations, the 10 mL sample was concentrated by centrifugation at 3,000 rpm for 10 min at room temperature. Nine milliliters of supernatant was then removed, the pellet resuspended, and the remaining 1 mL transferred to a microcentrifuge tube and centrifuged at 13,000 rpm for 5 min at room temperature. After centrifugation, 800 µL of supernatant was removed, and the remaining pellet was suspended in the remaining 200 µL of solution and transferred to the corresponding tube with the filter for later DNA extraction and was frozen at −20°C. DNA was extracted using the MasterPure complete DNA purification kit (Epicentre Biotechnologies Inc., Madison, WI, USA) following the manufacturer’s instructions.

#### Quantitative PCR

DNA samples were analyzed using the Applied Biosystems QuantStudio 6 Flex Fast Real-Time PCR system (ThermoFisher, Waltham, MA, USA). The TaqMan qPCR assay was performed for *Legionella* spp. and *L. pneumophila* targeting the 16S rRNA gene and for *P. aeruginosa* targeting the extracytoplasmic function sigma factor, ecfX, gene as described previously (36, 44). Data are expressed as genomic copies (GC) per L. The limit of detection for each qPCR assay was 1.4 log10 GC L^−1^. The threshold cycles (Ct) were calculated using QuantStudio Software v.1.6.1 (Waltham, MA, USA).

#### Scanning electron microscopy and energy-dispersive X-ray spectroscopy

For the DSS experiment, PVC and Cu coupons (Fig. 2) were imaged by scanning electron microscopy (SEM) and energy-dispersive X-ray spectroscopy (EDS) to image the biofilm and determine the fate of Cu and Ag ions within the system. Two coupons (one Cu and one PVC) were sampled prior to the addition of Cu and Ag ions, and four coupons (two Cu and two PVC) were sampled at the conclusion of the Cu and Ag addition. Coupons were analyzed at EPA’s Advance Materials and Solids Analysis Research Core (AMSARC) in Cincinnati, OH. The coupons were examined using a JEM7600FE scanning electron microscope (JEOL USA, Inc. Peabody, MA). The elemental composition of particles was identified using both an Oxford X-Max 50 EDS (Oxford Instruments America, Inc., Concord, MA) and a low-angle electron backscatter detector. The EDS spectra were analyzed using Aztec Software (Oxford Instruments America, Inc., Concord, MA) (45).

### Statistical analyses

For the bench-scale experiments, paired *t*-tests were used to determine significant differences in ion concentrations from the beginning and end of the experiments. Repeated measures ANOVA was used to determine the significance of time (defined as timepoints analyzed) and treatment (defined as experimental treatment, e.g., “Cu treatment” as described above) and their interaction (time × treatment). If a significant treatment and/or time effect was observed, pairwise comparisons were made with a Bonferroni correction with *t* = 0 min being the reference group for the bench-scale experiments.

For the pilot-scale experiments, Spearman rank correlation analyses were used to determine significant correlations between our water quality parameters (such as Cu and Ag ion concentration, pH, and temperature) and colony counts from our various media types. Data were evaluated for normality, and nonparametric tests were used if needed. Significance was determined at a level of 0.95. Statistical analyses were conducted in R v 4.1.3 (46) and RStudio 2022.02.0+443.

## RESULTS

### Bench-scale experiments

**Fig 3:**
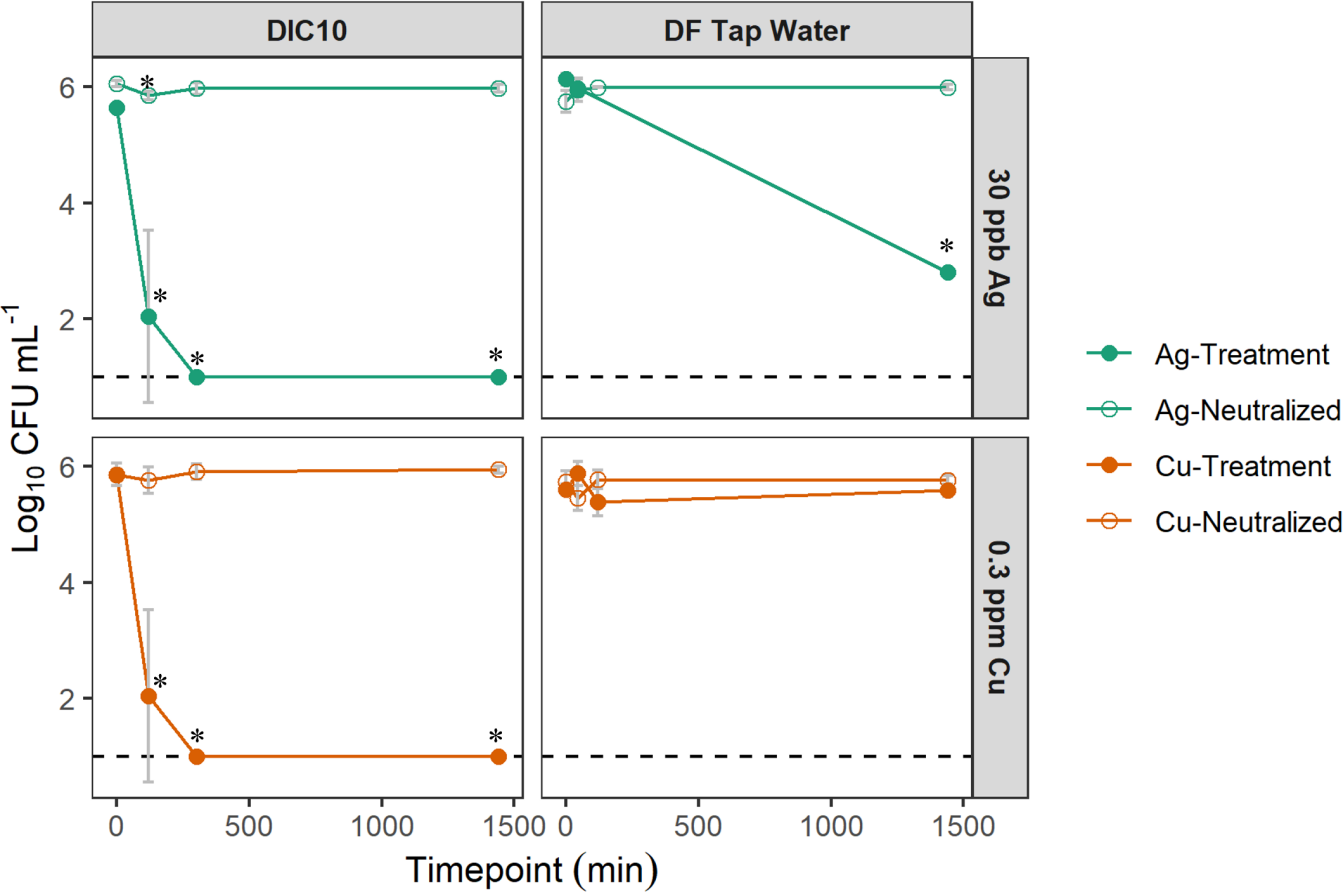
Lp inactivation during the benchtop experiments in DIC10 and DF Tap Water. Lp was exposed to Ag (green circles) and Cu (orange circles) in either DF Tap Water (right panels) or DIC10 (left panels). The neutralized treatments (open circles, top and bottom panels) represent treatments where the bactericidal activity of Cu and Ag was neutralized for the duration of the treatment. Points represent the mean ± SD of four replicates. Bench-scale experiments were conducted on one occasion. The dashed line represents LOD. Asterisks denotes significance at the 0.05 level.

We successfully reached our target ion concentrations for both Cu and Ag (Table S1). Ion concentrations were not significantly different between the start and end of the experiment (Cu: *t* = −2.9, df = 3, *P* = 0.06 and Ag: *t* = −1.7, df = 3, *P* = 0.18). There was no significant difference between dissolved and total ion concentrations (*t =* −2.2, df = 7, *P* = 0.06). In the DIC10 experiments, we observed a 3.5–4 log_10_ reduction by 120 min (2 h) and complete Lp inactivation by 300 min (5 h) in the Cu treatment and the Ag treatment (Fig. 3, left panels). For Cu-treated Lp (i.e., Cu treatment) in DIC10, a significant effect was observed for treatment (*F_1,12_* = 277.5, *P* < 0.005), time (*F_3,12_*, 20.8, *P* < 0.005), and treatment × time (*F_3,12_* = 19.6, *P* < 0.005). A significant decrease in Lp concentration was observed at every timepoint within the Cu treatment in DIC10 when compared to *t* = 0 timepoint (Fig. 3, bottom left panel). In the Ag treatment in DIC10, a significant effect was observed for treatment (*F_1,12_* = 321.9, *P* < 0.005), time (*F_3,12_* = 22.1, *P* < 0.005), and treatment × time (*F_3,12_* = 29.7, *P* < 0.005). And a significant decrease in Lp concentration was observed at every timepoint within Ag treatment (Fig. 3, upper left panel) in DIC10.

However, for experiments conducted in sterilized tap water (or DF Tap Water), Lp inactivation was only observed in the Ag treatment and at a much slower rate with an observed 3.3 log_10_ reduction by 1440 min (24 h) (Fig. 3, upper right panel). In the DF Tap Water experiments using Cu, neither treatment (*F_1,20_* = 1.2, *P* = 0.28) nor time (*F_3,20_* = 0.91, *P* = 0.45) had a significant impact on Lp concentration. And no decrease in Lp concentration was observed at any timepoint during Cu treatment (Fig. 3, bottom right panel). In the DF Tap Water experiments using Ag, a significant effect was observed for treatment (*F_1,17_* = 342.9, *P* < 0.005), time (*F_3,17_* = 320.7, *P* < 0.005), and treatment × time (*F_2,17_* = 423.9, *P* < 0.005). A significant decrease in Lp concentration was observed at 1440 m timepoint within the Ag treatment (Fig. 3, upper right panel). For the experimental controls, sodium thiosulfate was shown to neutralize the bactericidal activity of the Cu and Ag at all timepoints (Cu neutralized and Ag neutralized in Fig. 3 open circles) in both the DIC10 and DF Tap Water experiments. Data from our control and neutralized treatments are presented in Fig. S1, which also demonstrates that the sodium thiosulfate neutralizer did not negatively impact Lp growth.

### Pilot-scale experiments

#### Electrolytic production of ions

A commercially available CSI unit was installed and tested in the DSS. However, the manufacturer’s recommended levels of each ion (0.4 ppm for Cu and 0.04 ppm for Ag) could not be achieved even after several weeks of operation and troubleshooting with the company’s engineering team (Table S2). Lp was detected via qPCR at the Tank and Loops A and C during this phase.

#### Dissociation experiment

**Fig 4:**
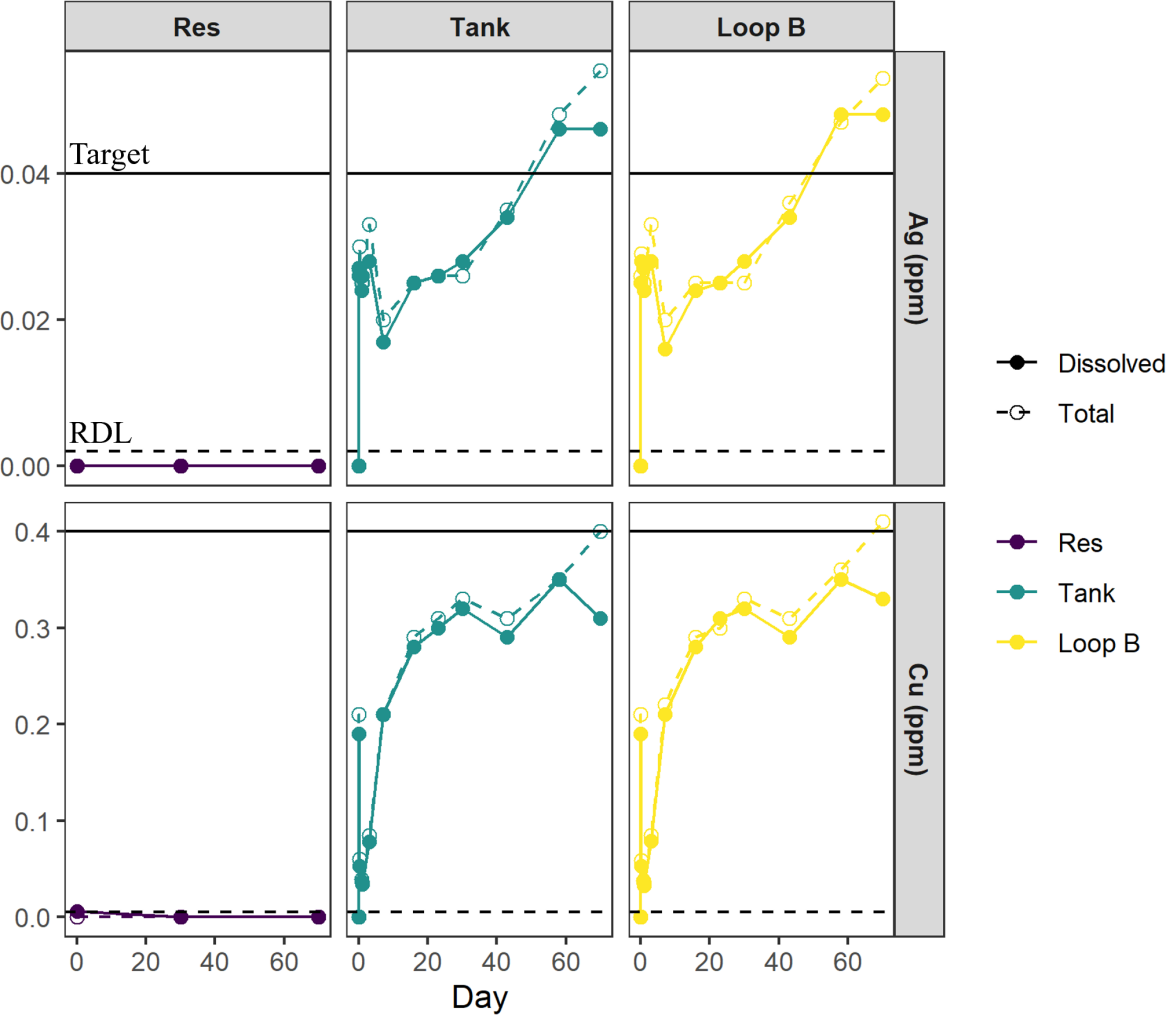
Dissolved (solid circles) and total (open circles) ion concentrations for Ag (top row) and Cu (bottom row) for the Res, Tank, and Loop B sites within the DSS dissociation experiment. The bottom dashed line represents the reporting detection limit in each panel, and the top solid line represents the target concentration for each ion.

We successfully reached our target concentrations for Cu and Ag ions using dissociation (Fig. 4) in the DSS. At the Tank and Loop B sampling locations, Cu ranged from 0.00 ppm at the beginning of the experiment to 0.41 ppm at the end of the experiment. Ag concentrations ranged from 0.00 ppm at the beginning of the experiment to 0.054 ppm at the end of the experiment, respectively. However, it took time (approximately 70 days for Cu and 43 days for Ag) to reach our target concentrations and the flow rate of Cu and Ag ions into the DSS needed to be monitored and adjusted throughout the duration of the experiment (see Fig. S3 for biofilm images). Total and dissolved concentrations of Cu and Ag were significantly different (Cu: *Z* = 19.5, *P* < 0.005; Ag: *Z* = 46, *P* = 0.02) with dissolved concentrations being less than total concentrations across all timepoints. Cu and Ag were not detected in the supply reservoir (site Res) (Fig. 4), confirming that Cu and Ag detected in the loop were a result of our experiment.

Concentrations of Cu and Ag ions were comparable between the ICP analysis and Hach colorimetric analysis (Fig. S2), with the latter providing a quick and useful indicator of ion levels when an immediate measurement of ion concentration was needed. However, ICP analysis had a lower detection limit and gave more precision, especially for the Ag analysis; thus, ICP results are discussed here.

**Fig 5:**
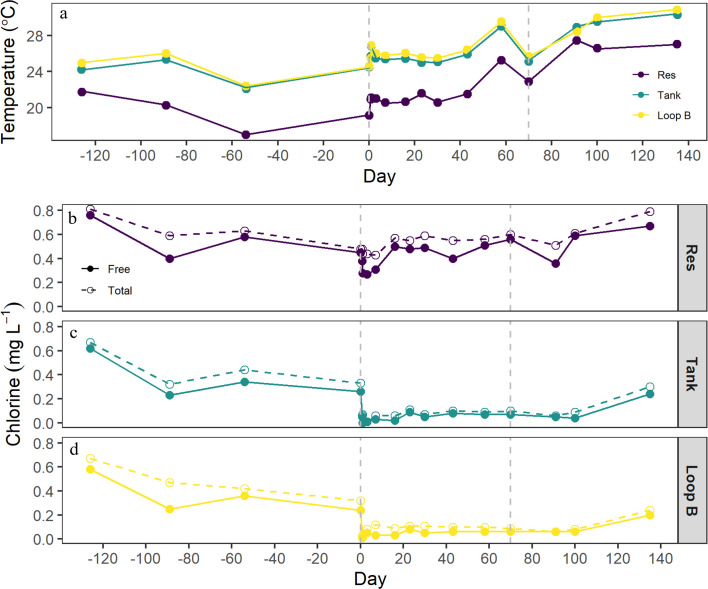
Water temperature (panel a), free chlorine (solid circles, panels b, c, and d), total chlorine (open circles, panels b, c, and d) for the Res, Tank, and Loop B sites within the DSS dissociation experiment. The vertical dashed lines represent the duration of the Cu and Ag treatment (days 0–70).

Various water quality parameters monitored during the DSS dissociation study are presented in Table 1. Overall, water temperature within the DSS ranged from 17.0°C to 30.9°C. The Res site was consistently cooler (mean 22.1°C) than the rest of the DSS, while temperature within the loop itself was warmer (27°C) but also consistent (Fig. 5a). The increases in temperature from day 0 to 135 throughout the DSS (20°C–24°C) were most likely due to seasonality (day 0 was in March, while day 135 was in July, which corresponds to the cooler and warmer seasons, respectively, in the United States). Overall, free and total chlorine levels were low throughout the monitoring period (<1 mg L^−1^) (Fig. 5b through d). Upon the start of the Cu and Ag treatment (day 0 in Fig. 5) and for the duration of the Cu and Ag treatment (day 70), free and total chlorine levels were <0.2 mg L^−1^ at the Tank and Loop B sites (Fig. 5c and d). This drop in chlorine was not observed at the Res site which supplies water to the DSS. Chlorine levels did not return to levels observed prior to the Cu and Ag treatment at the Tank and Loop B sites until day 135 (or approximately 2 months) after the conclusion of the Cu and Ag treatment (Fig. 5c and d). Free and total chlorine was significantly negatively correlated to water temperature (*r* = −0.72, *P* < 0.005; *r* = −0.69, *P* < 0.005, respectively).

**TABLE 1:** Various water quality parameters for the DSS pilot loop study including background monitoring^*a*^

Parameter	Res	Tank	Loop A	Loop B	Loop C
Free Cl_2−_ (mg L^−1^)	0.47 (0.27, 0.76)	0.13 (0, 0.62)	0.13 (0.02, 0.57)	0.13 (0.01, 0.58)	0.13 (0.01, 0.65)
Total Cl_2−_ (mg ^L−1^)	0.57 (0.43, 0.81)	0.17 (0.01, 0.67)	0.18 (0.02, 0.71)	0.18 (0.03, 0.67)	0.19 (0.03, 0.71)
Water temperature (°C)	22.1 (17.0, 27.5)	26.1 (22.2, 30.4)	26.4 (22.2, 31.0)	26.5 (22.4, 30.9)	26.5 (22.5, 31)
Water hardness (mg L^−1^)	138 (120, 154)	138 (120, 154)	137 (120, 154)	136 (120, 154)	136 (120, 154)
TOC (mg L^−1^)	-	-	1.07 (0.14, 1.69)	-	-
Conductivity (µS cm^−1^)	334 (304, 389)	-	-	-	-
ORP (mv)	385 (287, 623)	-	-	-	-
pH	8.44 (8.17, 8.65)	-	-	-	-

^*a*^ TOC, conductivity, ORP, and pH were only collected at one location in the DSS and only collected during the Cu and Ag treatment. Values are mean (min, max). -, no sample was collected for that site.

The presence of Cu^2+^ may enhance the formation of DPBs (30), but in this study, the concentration of DPBs did not exceed maximum contaminant levels (MCLs) set forth by the US EPA (40 C.F.R. § 141, 2024). Specifically, total trihalomethanes and total haloacetic acids (HAA5) were measured during the Cu and Ag treatment. TTHMs were quantified at 35.2 ug L^−1^ and 15.9 ug L^−1^ on day 30 at the Res and Tank sites, respectively. And at day 70, TTHMs were quantified at 37.2 ug L^−1^ and 14.9 ug L^−1^ in the Res and Tank sites, respectively. At day 30, HAA5s were quantified at 10.4 ug L^−1^ and 23.2 ug L^−1^ in the Res and Tank sites, respectively. At day 70, HAA5s were quantified at 9.3 ug L^−1^ and 22.2 ug L^−1^ in the Res and Tank sites, respectively.

**Fig 6:**
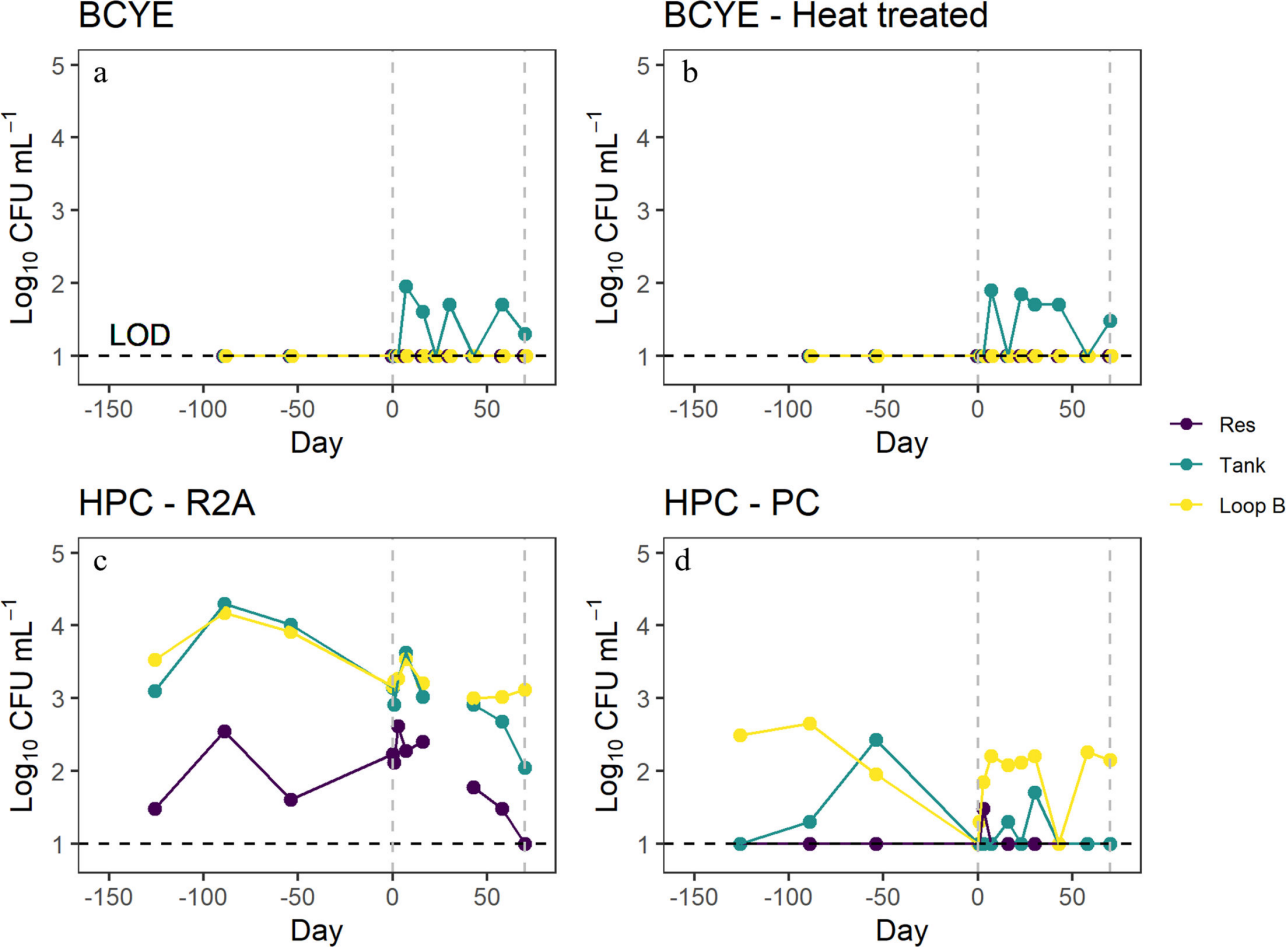
CFU mL^−1^ for various media types from the Res, Tank, and Loop B sites within the DSS dissociation experiment. The vertical dashed lines represent the duration of the Cu and Ag treatment (days 0–70). BCYE and BCYE heat-treated CFU values are only for *Legionella.* The horizontal dashed line represents the limit of detection.

**Fig 7:**
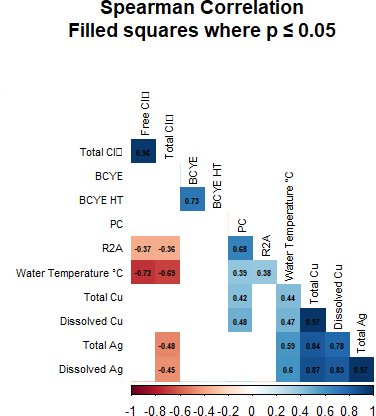
Spearman correlation matrix for various water quality parameters and log_10_ CFU mL^−1^ counts for various media types. Values correspond to the correlation between two variables (ranging from −1 to 1). Only significant correlations are shown (*P* ≤ 0.05).

Previous work has shown faster Lp inactivation as Cu and Ag concentrations increased (12). Therefore, we expected to see a negative correlation between microbial CFU counts and increasing Cu and Ag concentrations, but across all plate enumeration methodologies (BCYE, BCYE HT, R2A, and PC), no significant negative correlations were observed between colony counts and Cu or Ag ionization treatment (days 0–70 in Fig. 6 and 7). Lp was identified during the Cu and Ag ionization treatment (Fig. 6a and b), and colony identification was confirmed by qPCR. Lp colonies enumerated from BCYE agar were only identified at the Tank site (Fig. 6a and b) and varied from 0 CFU/mL to 90 CFU/mL throughout the duration of the Cu Ag treatment. Heterotrophic bacteria (as determined via HPCs using both PC and R2A medium) were present in all sites with the Res site containing lower quantities throughout the monitoring period (Fig. 6c and d). A positive correlation was observed between the PC media and concentrations of total Cu (*r* = 0.42, *P* = 0.04; Fig. 6) and dissolved Cu (*r* = 0.48, *P* = 0.02; Fig. 7). A significant positive correlation was observed between water temperature and the two HPC media (PC: *r* = 0.39, *P* = 0.007 and R2A: *r* = 0.38, *P =* 0.02) (Fig. 7).

**Fig 8:**
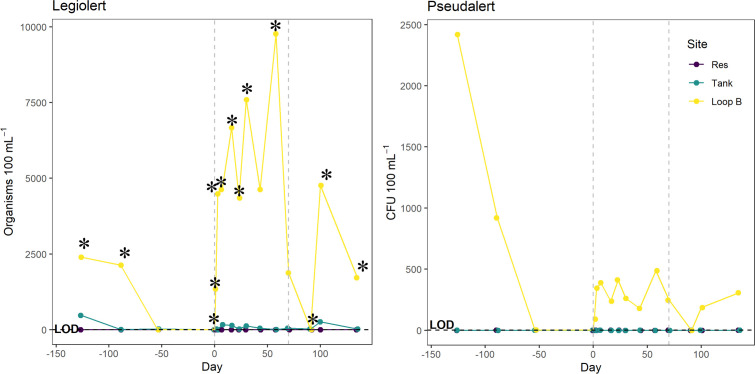
Legiolert and Pseudalert counts from the Res, Tank, and Loop B sites within the DSS dissociation experiment. The vertical dashed lines represent the duration of the Cu and Ag treatment (days 0–70). Asterisks (*) denote false positives detected in wells of the Legiolert tray.

Although Lp CFU was not detected at site Loop B, Loop B routinely tested culture positive for Lp according to the Legiolert test kits (Fig. 8), and for most sample dates (9 out of 16), positive Lp readings were above the method detection limit of 2272.6 MPN 100 mL^−1^ (Fig. 8). However, upon sampling of randomly selected positive wells (indicated by a dark brown color), wells were found to be false positives 86% of the time in Loop B as determined by PCR. Some of the positive wells were streaked onto BCYE agar and incubated. A colony pick was also determined to be negative for Lp via qPCR. The identity of the organism that caused the false positive was determined to be *Pseudoxanthomonas indica* via 16S rRNA gene sequencing targeting the V3–V4 region. True positives for Lp were detected from the Tank site using the Legiolert test kits. For *P. aeruginosa*, Loop B routinely tested positive and ranged from 1 to 2419.6 CFU mL^−1^, while the Tank and Res site were *P. aeruginosa* culture negative (Fig. 8) using the Pseudalert test kits. Positive wells in the Pseudalert test kits were confirmed by colony lysate *P. aeruginosa* qPCR 100% of the time.

**Fig 9:**
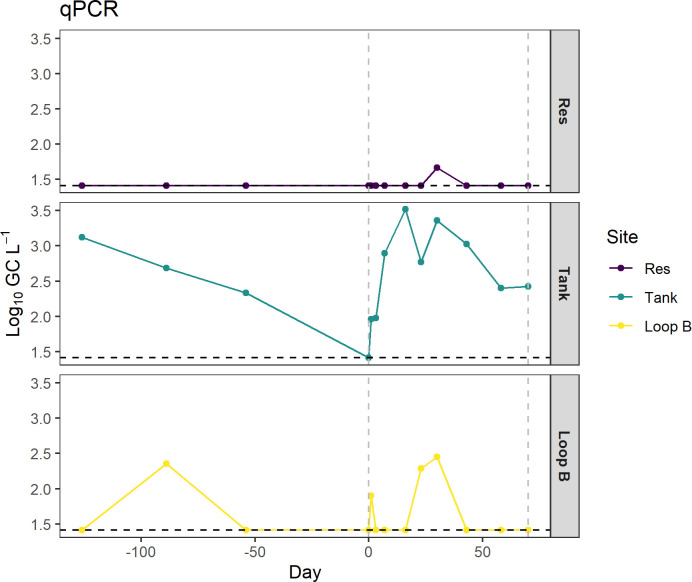
Gene copies from the qPCR Lp assay for the Res, Tank, and Loop B sites within the DSS dissociation experiment. The horizontal dashed line represents the LOD for the qPCR assay. The vertical dashed lines represent the duration of the Cu and Ag treatment (days 0–70).

From our qPCR analysis, Lp was detected in the bulk water at all sites (Fig. 9). The Tank site tested positive on 12 of 13 sampling dates (~92%), and Loop B tested positive on 4 of 13 sampling dates (~31%). During Cu Ag treatment, Lp was detected in all samples at the Tank site, while Lp detection was intermittent at the Loop B sampling location (Fig. 9). There were no significant correlations between GC L^−1^ and either dissolved or total Ag concentrations, while significant positive correlations were observed between GC L^−1^ and dissolved and total Cu concentrations (*r* = 0.32, *P* = 0.02; *r* = 0.30, *P* = 0.3, respectively).

## DISCUSSION

We evaluated the effectiveness of Cu and Ag ions at both the bench and pilot scale using a dissociation method that had not been previously demonstrated at the pilot scale. Our findings highlight the importance of having a comprehensive understanding of a system’s water chemistry prior to utilizing Cu and Ag ions as a tool to manage Lp.

In our bench-scale experiments, we identified independent levels of Cu and Ag that were effective in inactivating Lp in an experimental buffer (DIC10) and compared the results to experiments conducted with dechlorinated sterile tap water. We found that both 0.3 ppm Cu and 0.03 ppm Ag inactivated Lp in 5 h in DIC10—a solution that contained 10 mg L^−1^ of DIC and had a pH of 8. Properties of the DIC medium allowed Cu and Ag ions to stay in solution and likely contributed to the bactericidal capabilities of these ions. Conversely, when the same experiments were conducted in dechlorinated, filter sterile tap water (DF Tap Water) (pH of 8.2–8.3 and average carbon content of <1 mg L^−1^), only Ag was effective in inactivating Lp albeit at a much slower rate (i.e., complete inactivation did not occur within 24 h, Fig. 3, top panels). Here, we have identified independent concentrations of Cu and Ag which are efficacious when previously these ions have primarily been tested in conjunction with one another (but see references 13 and 47 for exceptions). However, the differing inactivation rates between these two experiments highlight the importance of having prior knowledge of a system’s water chemistry when deciding if Cu and Ag ions are an appropriate solution. Complexation of Cu and Ag likely reduced the effectiveness within the DF Tap Water. Prior work has shown that the biocidal activity of Cu^2+^ is hindered at pH >7 and higher carbon contents (>5 mg L^−1^ organic carbon) (14, 28), yet a pH of >7 for drinking water is common and, hence, demonstrates the importance of evaluating this methodology under conditions typical of drinking water. Similarly, we observed that the antimicrobial properties of Ag were not impacted by higher pH in our bench-scale experiments (14). Cu-amended point-of-use (POU) carbon filters were ineffective at removing Lp from tap water that had a pH of >7 when compared to control POU filters; however, POU filters that were amended with both Cu and Ag reduced Lp from effluent (27). This could suggest that Ag is the primary contributor of biocidal activity when pH >7 and lower carbon content as observed in the bench-scale experiments (Fig. 3). However, POU filters amended with Ag alone were not evaluated in Molloy and co-author’s (27) work. In our bench-scale experiments, testing Cu and Ag independently of each other in different experimental buffers allowed us to elucidate the potential impacts water chemistry can have on using Cu and Ag ions as a tool in managing Lp. Effective levels of copper and silver ions vary within the literature and range from 0.1 to 0.8 mg L^−1^ or parts per million (ppm) for Cu and 0.012–0.08 mg L^−1^ or ppm for Ag, and our study highlights that the varying efficacious concentrations may result from the variation that exists in building water chemistries (see reference (48) for a summary table on different levels tested across studies at the building scale).

We demonstrated that dissociation can be used as a method to produce Cu and Ag ions in a large pilot-scale pipe loop, and this methodology can successfully meet and maintain target concentrations of metal biocides. We hypothesized that Cu and Ag ions would have a negative impact on the microbial community, but, generally, we did not see an impact of the Cu and Ag treatment on the microbial parameters we measured (Lp on BCYE, HPCs on R2A and PC, Legiolert, Pseudalert, and Lp from qPCR from concentrated water samples) during the 10-week dissociation treatment. During this experiment, Lp was detected in the bulk water at all sites within the DSS via qPCR, while culturable Lp was only identified at one site, the Tank. The disagreement between these methodologies likely results from the nature of the methodology, i.e., qPCR does not differentiate between various forms of Lp (live, dead, and viable but not culturable), while culture methods would only quantify live cells. We observed that Lp concentrations were the highest, but varied, in the Tank throughout the duration of Cu and Ag dissociation treatment. Although the Tank was the site of the Cu and Ag addition, others have also observed no impact of Cu and Ag ions on Lp levels (16, 21, 24). A limitation of our work is that biofilms and free-living amoeba (FLA) were not sampled, but future work could look at the impact of Cu and Ag ions on biofilm and FLA communities. Biofilms may be especially relevant as we observed Cu and Ag bound in biofilm in the images of Cu and PVC coupons.

Monitoring of ion levels is essential when utilizing CSI units given how variable generated ion concentrations can be within premise plumbing systems (26). Using dissociation allowed precise dosing of the DSS because the volume, refresh rate, and other factors were known; however, considerable time was required to reach the target concentrations for both Cu and Ag while not exceeding the MCL of 1.3 mg L^−1^ for Cu and SMCL of 100 µg L^−1^ for Ag. This delay likely resulted from ion interactions with pipe materials and biological material and impact of water chemistry on dissolved ion concentrations within the DSS which was observed during a previous building-scale evaluation of Cu and Ag ions (26). SEM images and EDS spectra demonstrated that Cu and Ag were in the biofilm on the Cu and PVC coupons at the conclusion of the Cu and Ag addition (Fig. S3). Utilizing a dissociation methodology would be more challenging to implement at a larger building scale where water usage is more complex and highly variable. The dissociation approach would require more routine monitoring and adjustments of ion levels, stock solutions, and dosing. However, commercial CSI units (which use an electrolytic method) also need to be frequently monitored for scaling, power level, condition of the consumable metal bars (ion source), and ion levels delivered at the source and various distal sites (22, 24, 26).

Water chemistry is an important determinant of the antimicrobial properties of Cu and Ag. We observed Cu precipitates at the outflow of the Cu injection line, and this likely influenced the bactericidal properties of Cu in our experiment. While the use of Cu^2+^ can be suggested as an Lp control mechanism, TOC levels and pH observed in the DSS (TOC = 1.07 mg L^−1^, pH range = 8.17–8.65) are typical of drinking water and have been shown to be outside the range at which Cu^2+^ will stay in solution (14, 28, 29). Therefore, the application of Cu should be revisited under these conditions. Additionally, chlorine residual decreased after initiating the Cu and Ag treatment, and it is unclear if the decrease was due to a lack of refresh water during the first 24 h of the treatment or if there was additional chlorine demand exerted by the Cu^2+^ ions. Similar chlorine decay can be significantly enhanced in the presence of Cu and at pH levels observed in the DSS (49). Future work could incorporate the direct measurement of Cu^2+^ ions which could better illustrate Cu speciation within a system (28).

The effectiveness of Cu and Ag ions in controlling Lp in premise plumbing systems is unclear. Bench-scale studies have found that Cu and Ag can be effective in the inactivation of Lp (12–14) and enteric bacteria (50). At the pilot scale, Cu and Ag have been found to be initially effective, but regrowth of Lp and other waterborne pathogens was observed for both planktonic and biofilm-associated stages (11, 15). At the building scale, the disinfection efficacy of Cu and Ag ions has been inconsistent ranging from complete eradication of Lp (20), decreases in Lp-positive sites (e.g., taps) (22, 24, 31, 33, 34, 51), initial success followed by Lp regrowth (23), to no change in Lp detection (16, 21). Lp monitoring in building-scale studies has varied in duration (from 4 months to 8 years), and the effectiveness of Cu and Ag ions has been observed to change over time in some studies. The use of Cu and Ag ions can also be effective in the control of other waterborne pathogens such as *P. aeruginosa*, *Stenotrophomonas maltophilia*, and *Acinetobacter baumannii* (11, 47, 50), but we did not observe that here when *P. aeruginosa* was evaluated.

Legiolert test kits were used to quantify Lp within the DSS, but false positives were routinely found at site Loop B. False positives were confirmed by performing PCR on well contents from the Legiolert trays and on colony isolates cultured from the same well contents. If Legiolert is the only detection method used, concentrations of Lp could be overestimated and, thus, would require corrective actions by premise plumbing operators resulting in unnecessary costs, actions, and public health alarm. While Legiolert test kits claim specificity for Lp, previous work has also found false positives with these kits (35, 52). It has been suggested that there may be cross-reactivity with the enzyme-substrate methodology when other Gram-negative bacteria are present in samples (52). The organism identified in this study (*Pseudoxanthomonas indica*) is a Gram-negative bacterium and had not previously been known to cause false positives in Legiolert test kits. This result is worth reporting given the wide use of these test kits and the previous unidentified organism which led to a false positive. The false positives detected here also highlight the importance of using various methods to identify Lp.

The results from this study indicate that pH and TOC can influence the effectiveness of Cu and Ag on Lp inactivation; however, other water quality parameters and factors may limit applications of Cu and Ag biocides for controlling premise plumbing pathogens. For example, Song and coauthors (28) reported that PO_4_^3−^ can influence the biocidal activity of Cu. Water hardness has been observed to affect the toxicity of metals, with toxicity decreasing as hardness increases (53). We did not directly test the impact of water temperature on the efficacy of this methodology, but temperatures observed in the DSS varied from 17℃ to 30°C, and future work could evaluate the direct impact water temperature has on the efficacy of Cu and Ag ionization. We observed that Ag alone is effective at Lp inactivation (at the bench scale) and may be less impacted by pH, temperature, and carbon levels, and, therefore, the use of Ag alone needs further examination. In this study, target levels of Cu and Ag ions were difficult to reach, and the effectiveness of Cu and Ag ions was likely hindered at the pilot scale, but it remains to be seen if lower levels of ions can be effective when combined with other control mechanisms such as superheat-and-flush, ozonation, or UV radiation (7). Lastly, we did not evaluate the impact of Cu and Ag ionization on various strains and serogroups of Lp, and future work could explicitly evaluate the impact of this methodology on differing Lp strains and serogroups as previous work has suggested that Lp may become resistant to the bactericidal capabilities of Cu and Ag (15, 17, 23).

### Conclusions

While positive experiences using Cu and Ag ions to control Lp have been reported, these reports often omit values of important water quality parameters that allow potential users or managers to have a complete view of the premise plumbing system being evaluated and treated. These knowledge gaps hinder the ability of potential users or managers to make informed decisions about whether the use of Cu and Ag ions is an appropriate treatment option for a given premise plumbing system. In some cases, the use of Cu and Ag ions is found to be effective and is suggested as a tool to control Lp without sufficient evidence that Lp was ever a problem to begin with (20). In conclusion, the results presented here highlight the importance of prior knowledge of a system’s water chemistry in determining if Cu and Ag ions are an appropriate tool for managing Lp in premise plumbing systems.

## Data Availability

Data are publicly available through the U.S. EPA ScienceHub website at https://doi.org/10.23719/1531898.

## SUPPLEMENTAL MATERIAL

Supplemental material aem.01073-24-s0001.docx Figures S1 to S3; Tables S1 and S2.

ASM does not own the copyrights to Supplemental Material that may be linked to, or accessed through, an article. The authors have granted ASM a non-exclusive, world-wide license to publish the Supplemental Material files. Please contact the corresponding author directly for reuse.
