# Supplementary material for: Cu- and Ag-mediated inactivation of L. pneumophila in bench- and pilot-scale drinking water systems
Source: Appl Environ Microbiol. 2024 Dec 18;91(1):e01073-24. doi: 10.1128/aem.01073-24 (PMC11784313; doi:10.1128/aem.01073-24)
Supplement: Supplemental material — Figures S1 to S3; Tables S1 and S2. [file aem.01073-24-s0001.docx]

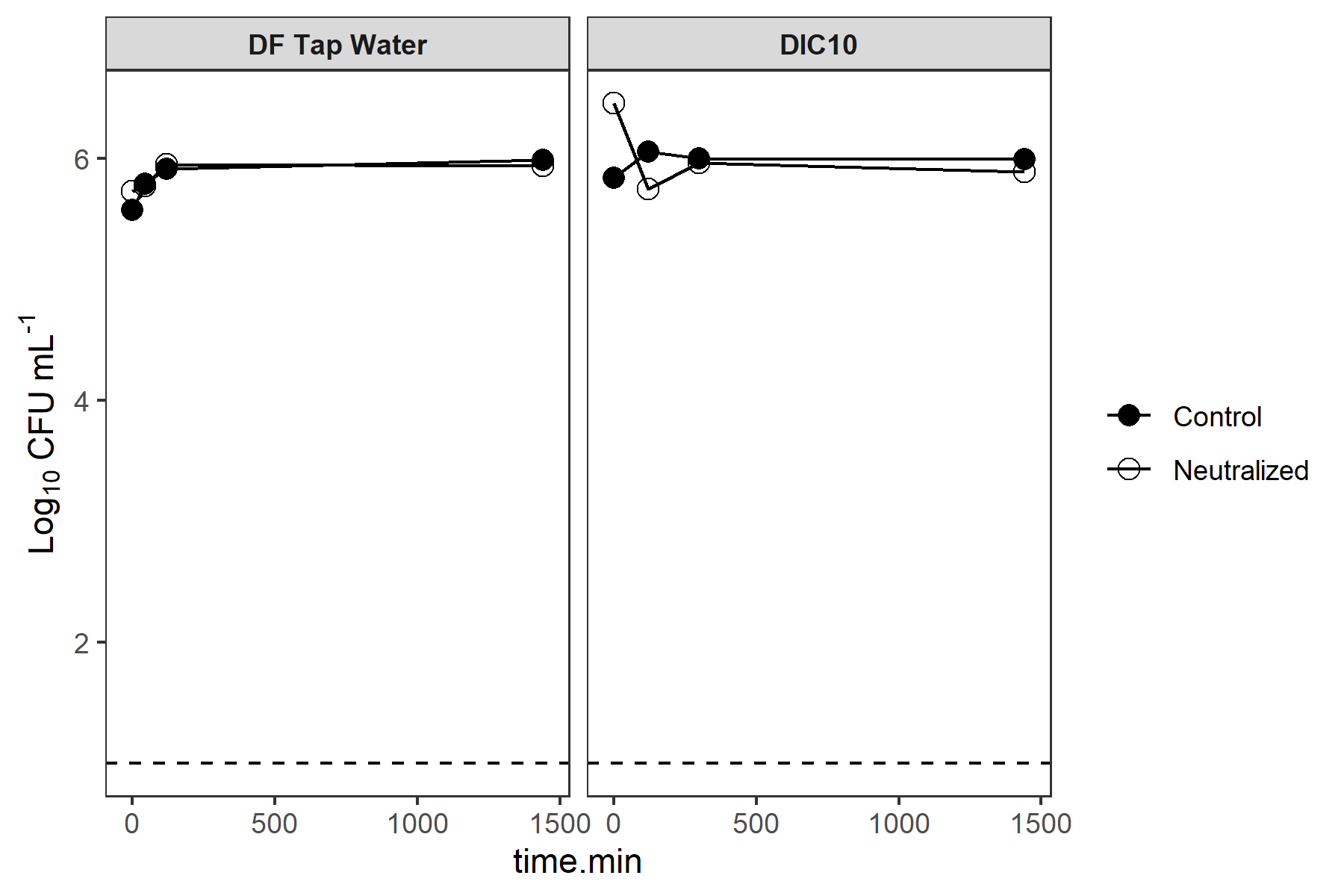
Supplementary Figures and Tables

Supplementary Figure 1: Results of the control and Neutralized treatments from the benchtop experiments in DIC10 and DF Tap Water. The control treatments contained Lp and the associated experiment buffer (filled circles in both panels) while the neutralized treatments contained Lp, the experimental buffer, and the neutralizer (10% sodium thiosulfate). The dashed line represents LOD.

Supplementary Figure 2: Cu and Ag concentrations for the Tank and Loop A sites during the DSS dissociation study. Dissolved (solid circle) and Total (open triangle) concentrations were determined via ICP-MS analysis while Total concentrations (crosses) were determined via Hach kits (crosses) as an immediate determination of ion concentrations.


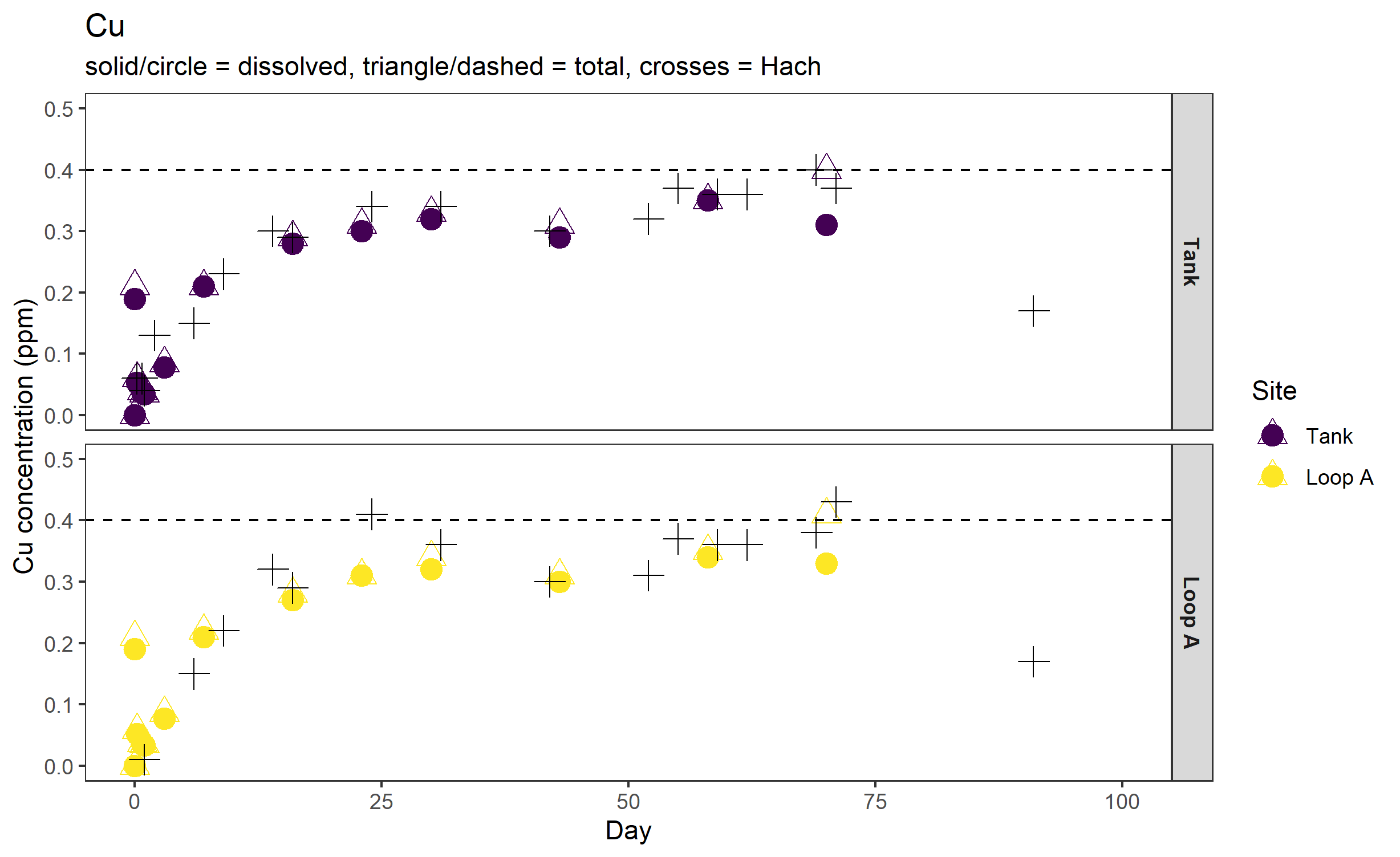

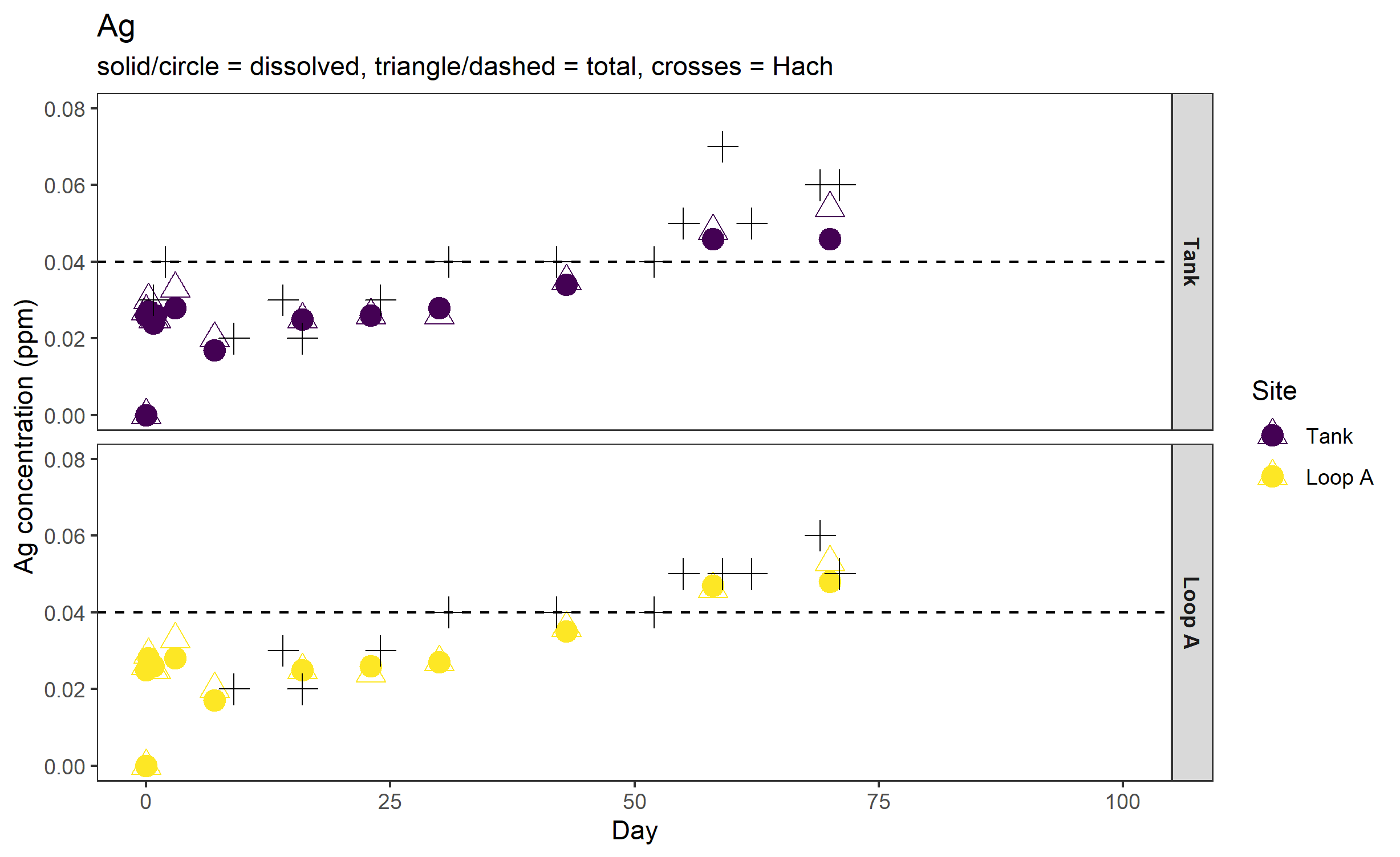


1. A PVC coupon from the DSS prior to Cu and Ag addition. SEM image (top image) and spectrum (bottom image). Spectrum is for the entire SEM image.


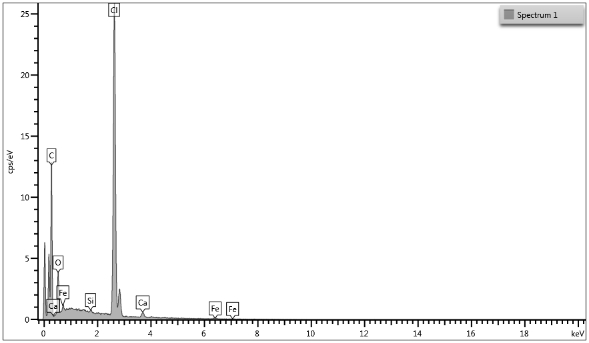

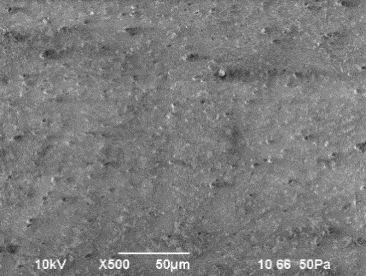

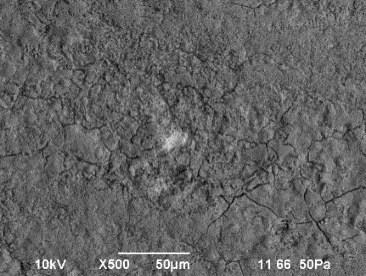

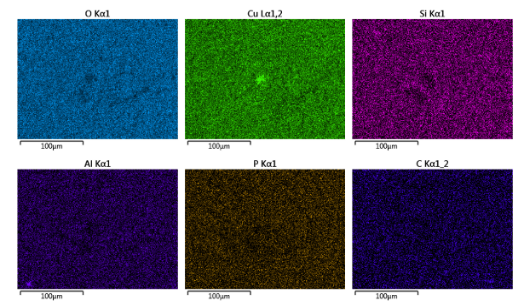


1. A Cu coupon from the DSS prior to the Cu and Ag addition. SEM image (top image) and spectrum (bottoms images). Spectrum correspond to the entire SEM image.


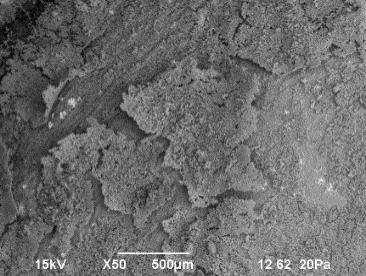

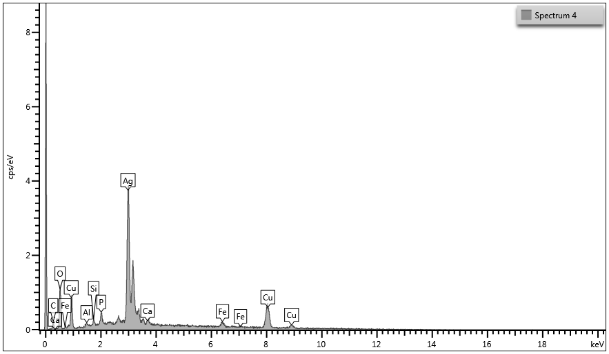


1. A Cu coupon from the DSS at the conclusion of the Cu and Ag addition. SEM images (top image) and spectrum (bottoms image). The spectra corresponds to the yellow box in the SEM image.


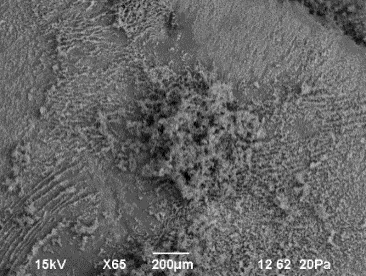

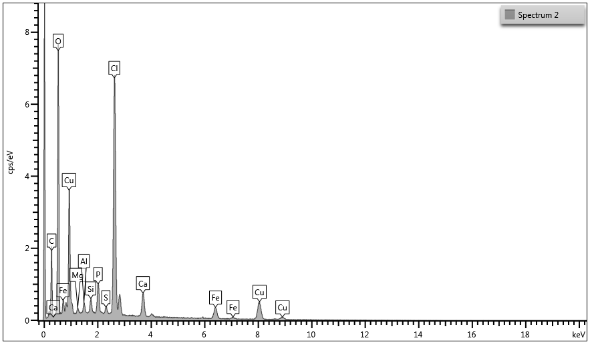


1. A PVC coupon from the DSS at the conclusion of the Cu and Ag addition. SEM image (top image) and spectrum (bottom image). Spectrum is for the entire SEM image.

Supplementary Figure 3 SEM images and EDS spectra for PVC (panels a and b) and Cu (panels c and d) coupons

Supplementary Table 1: Characteristics of the benchtop experiments conducted. Dissolved and total ion concentrations are presented from the beginning and end of the experiment. Ion concentrations were determined via ICP analysis.

|  | **Analysis** | **Beginning Ion Concentration** | **Ending Ion Concentration** | **Legionella Strain** | **Experimental Buffer** | **Timepoints (min)** |
| --- | --- | --- | --- | --- | --- | --- |
| Cu (target concentration: 0.3 ppm) | | |  |  |  |  |
|  | Total | 0.31 | 0.31 | Sg1-Oh | DIC10 | 0, 120, 300, 1440 |
|  | Dissolved | 0.29 | 0.28 | Sg1-Oh | DIC10 | 0, 120, 300, 1440 |
|  |  |  |  |  |  |  |
|  | Total | 0.36 | 0.35 | Sg1-Oh | DF Tap Water | 0, 45, 120, 1440 |
|  | Dissolved | 0.36 | 0.34 | Sg1-Oh | DF Tap Water | 0, 45, 120, 1440 |
|  |  |  |  |  |  |  |
| Ag (target concentration: 0.03 ppm) | | |  |  |  |  |
|  | Total | 0.022 | 0.022 | Sg1-Oh | DIC10 | 0, 120, 300, 1440 |
|  | Dissolved | 0.029 | 0.029 | Sg1-Oh | DIC10 | 0, 120, 300, 1440 |
|  |  |  |  |  |  |  |
|  | Total | 0.028 | 0.027 | Sg1-Oh | DF Tap Water | 0, 45, 120, 1440 |
|  | Dissolved | 0.030 | 0.029 | Sg1-Oh | DF Tap Water | 0, 45, 120, 1440 |

Supplementary Table 2: Total and dissolved concentrations of Cu and Ag in bulkwater from the DSS electrolysis experiment. Ions were produced via commercial CSI unit. Concentrations were determined via ICP-MS.

|  |  | **Timepoint** | | | | | | |
| --- | --- | --- | --- | --- | --- | --- | --- | --- |
| Cu  (Target = 0.4 ppm) | | **105 min** | **1 day** | **7 days** | | **37 days** | | |
| *Total* | | 0.057 ± 0.003 | 0.046 ± 0.011 | 0.037 ± 0.003 | | 0.3 ± 0.018 | | |
| *Dissolved* | | 0.052 ± 0.001 | 0.126 ± 0.153 | 0.034 ± 0.002 | | 0.273 ± 0.016 | | |
| Ag  (Target = 0.04 ppm) | |  |  |  |  | |  |  |
| *Total* | | 0.015 ± 0.001 | 0.016 ± 0 | 0.043 ± 0.03 | | 0.024 ± 0.001 | | |
| *Dissolved* | | 0.014 ± 0 | 0.039 ± 0 | 0.008 ± 0.001 | | 0.021 ± 0 | | |
